# HER-3 surface expression increases in advanced colorectal cancer representing a potential therapeutic target

**DOI:** 10.1038/s41420-023-01692-8

**Published:** 2023-10-28

**Authors:** Emily Capone, Thordur Tryggvason, Ilaria Cela, Beatrice Dufrusine, Morena Pinti, Francesco Del Pizzo, Helga Sigrun Gunnarsdottir, Tommaso Grottola, Vincenzo De Laurenzi, Stefano Iacobelli, Rossano Lattanzio, Gianluca Sala

**Affiliations:** 1 Department of Innovative Technologies in Medicine & Dentistry, University “Gabriele d’Annunzio” of Chieti-Pescara, Chieti, Italy; 2 Center for Advanced Studies and Technology (CAST), University “Gabriele d’Annunzio” of Chieti–Pescara, Chieti, Italy; 3 Department of Pathology, Landspítali – The National University Hospital of Iceland, Reykjavik, Iceland; 4 Department of Bioscience and Technology for Food, Agriculture and Environment, University of Teramo, Teramo, Italy; 5 Department of Medical, Oral and Biotechnological Sciences, University “Gabriele d’Annunzio” of Chieti–Pescara, Chieti, Italy; 6 Surgical Oncology Unit, Casa di Cura Pierangeli, 65124 Pescara, Italy; 7 MediaPharma s.r.l., Via Colonnetta 50/A, Chieti, Italy

**Keywords:** Predictive markers, Cancer

## Abstract

HER-3 (also known as ErbB-3) is a human epidermal growth factor receptor tyrosine kinases family member, and its expression in CRC (colorectal cancer) tissues was previously associated with poor prognosis. In this study, HER-3 expression was analyzed by immunohistochemistry in two cohorts of early and advanced metastatic CRC patients. The first cohort included 180 patients diagnosed with CRC in absence of lymph nodes or distant metastases (Stage I and Stage II), while the second was obtained from 53 advanced metastatic CRC patients who developed synchronous (SM) and metachronous (MM) liver metastases. In the first early-stage CRC cohort, 86 out of 180 (47.8%) tumors showed membranous expression of HER-3, with a mean percentage of positive tumor cells of 25.7%; conversely, in advanced metastatic CRC primary tumors, HER-3 was detected in all specimens, with a mean percentage of positive tumor cells of 76.1%. Kaplan–Meier curves showed that in the advanced metastatic CRC group, patients with HER-3^high^ tumors had a significantly lower Cancer-Specific Survival (CSS) rate compared to patients with HER-3^low^ tumors (*p* = 0.021). Importantly, this worse CSS rate was observed only in the MM subgroup of patients with HER-3^high^ tumors (*p* = 0.002). Multivariate analysis confirmed that high HER-3 expression represents a significant and strong risk factor for death in patients developing MM liver metastases (Hazard Ratio = 64.9; 95% Confidence Interval, 4.7–886.6; *p* = 0.002). In addition, using a specific anti-HER-3 antibody-drug conjugate, named EV20/MMAF, we showed that HER-3 + CRC cells can be efficiently targeted in vitro and in vivo. Overall, this study confirms that surface HER-3 is highly expressed in CRC and reveals that HER-3 expression increases in metastatic CRC patients compared to early stage. Importantly, the results suggest that HER-3 has a prognostic and therapeutic value in patients developing MM liver metastases.

## Introduction

Colorectal cancer (CRC) is a leading cause of cancer-related deaths worldwide, and despite the improvements in early detection and treatment strategies, prognosis of CRC has never been satisfying, especially for patients developing distant metastatic lesions [1, 2]. Approximately 30–50% of distant CRC metastases occur in the liver, followed by the lung, and they are classified as synchronous or metachronous, depending on whether they are detected contextually or after the primary tumor removal, with approximately equal incidence [3–6]. From a clinical point of view the discovery of prognostic markers and novel therapeutic targets would certainly benefit CRC patients.

HER-3 (also known as ErbB-3) is a member of the epidermal growth factor receptor (EGFR) family of receptor tyrosine kinases, which also includes EGFR, HER-2, and HER-4. HER-3 receptor has been implicated in promoting cancer cell proliferation, survival, migration, invasion, and resistance to chemotherapy and targeted therapies [7, 8]. HER-3 functions as a co-receptor with other EGFR family members or other Tyrosine Kinase Receptors (RTK)s as such c-MET and IGFR, and activates downstream signaling pathways, mainly the PI3K-AKT and MAPK-ERK pathways, which are critical for cancer cell growth and survival [7, 8]. Moreover, HER-3 expression has been associated with poor clinical outcome, tumor recurrence, metastasis, and reduced overall survival in several solid tumors, including CRC [9–13].

HER-3 is also a promising therapeutic target in CRC. Strategies to inhibit HER-3 signaling include naked and conjugated monoclonal antibodies targeting the extracellular domain of HER-3, small molecule inhibitors of HER-3 tyrosine kinase activity, and combination therapies that target multiple EGFR family members. Recent preclinical studies have demonstrated the efficacy of HER-3 targeted therapies in CRC models, including xenograft and patient-derived tumor models [14, 15]. Moreover, early-phase clinical trials have shown promising results with HER-3 targeted therapies, alone or in combination with other agents, in patients with advanced CRC [16–18].

In the present paper, we evaluated the immunohistochemical expression and the prognostic value of HER-3 in a cohort of early-stage CRC and in a second set of advanced metastatic CRC patients of whom both the primary tumors and corresponding synchronous and metachronous liver metastasis were available.

Furthermore, using a potent and specific auristatin F-based anti-HER-3 Antibody-Drug Conjugate (ADC) developed by our group [19–21], we evaluated potential of HER-3 as therapeutic target in CRC.

## Results

### HER-3 expression and clinical outcome

**Fig. 1:**
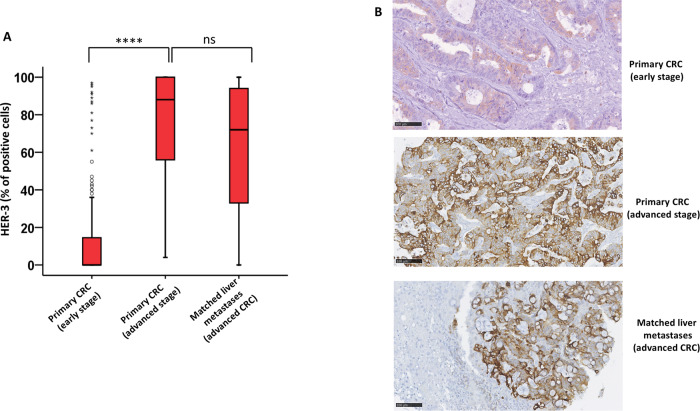
HER-3 expression in early and advanced/metastatic CRC patients. **A** The box-and-whisker plot displays the percentage of colorectal cancer cells stained with the anti-HER-3 antibody in all three specimen groups. The boxes’ upper and lower ends indicate the 75th and 25th percentiles, respectively. ****p < 0.0001 **B** HER-3 expression in primary CRC at early and advanced/metastatic stages and matched liver metastasis. Scale bar 100 μm.

HER-3 expression analysis was performed in early and advanced cohorts of patients. IHC analysis showed surface HER-3 expression in both groups. However, we found a dramatic increase of HER-3 expression in advanced metastatic setting compared to early-stage primary tumors. Indeed, while in early-stage CRC patients, only eighty-six out of 180 (47.8%) tumors showed membranous expression of HER-3, with an HER-3 positive tumor cells mean percentage ± SE of 25.7 ± 3.0 (range, 5 to 97%), conversely in the advanced metastatic CRC setting, all 53 primary tumors showed surface HER-3 expression (100%), with an higher HER-3 positive tumor cells mean percentage of 76.1% ± 3.8 (range, 4 to 100%). Regarding liver metastasis HER-3 expression, also in this case all but one liver metastases (98%) expressed HER-3, with a mean percentage of 63.0% ± 4.5 positive cells (range, 5 to 100%). Among the metastatic tissue samples, SM expressed HER-3 with a mean ± SE of 59.4% ± 7.7; MM with a mean ± SE of 65.2% ± 5.6 (Fig. 1A). Representative IHC images of HER-3 expression in all three groups are showed in Fig. 1B.

Then, correlation between HER-3 expression, clinicopathological characteristics and clinical outcome was performed. A receiver operating characteristic (ROC) curve was used to predict patient survival in order to determine the optimal cut-off value for HER-3 expression, considering the maximum Youden index in primary CRC tissues. For the first early-stage CRC group, the optimal cut-off value for HER-3 expression was 1%, with an area under the curve (AUC) of 0.440 (sensitivity = 38.1%, 1-specificity = 51.1%). Thus, tumors with ≥1% positive cells (*n* = 86) were classified as HER-3^Pos^. On the other hand, those that did not show any specific membrane staining for HER-3 (*n* = 94) were considered negative (HER-3^Neg^ tumors). For the advanced CRC group, the optimal cut-off value for HER-3 expression was 85%, with an area under the curve (AUC) of 0.569 (sensitivity = 63.0%, 1-specificity = 45.5%).

**Fig. 2:**
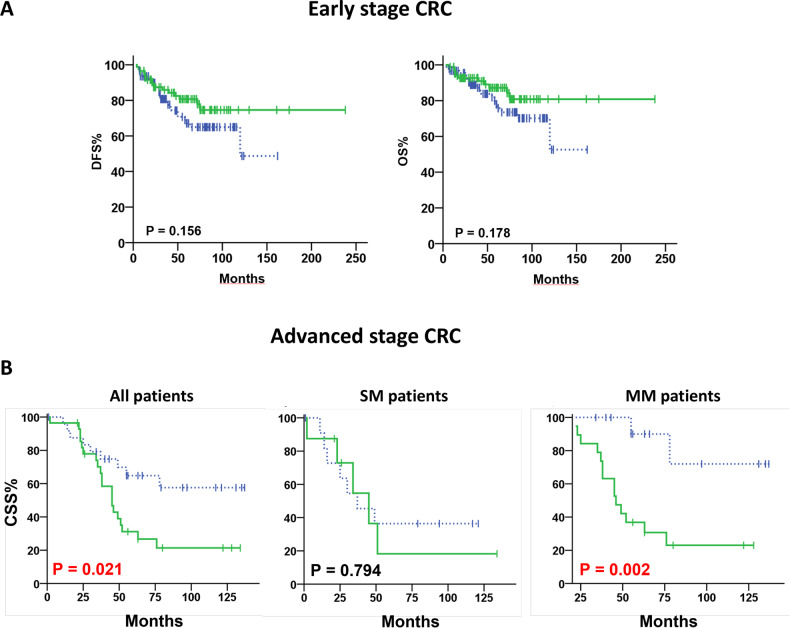
Correlation of HER-3 expression with patient outcome. **A** Kaplan–Meier curves for disease-free survival (DFS) and overall survival (OS) in patients with primary colorectal cancer tumors, grouped by high HER-3 expression (solid green line) and low HER-3 expression (dashed blue line). **B** The Kaplan–Meier plot shows the cancer-specific survival (CSS) curves for high and low HER-3 expression in primary colorectal cancer tumors. The solid green line represents high HER-3 expression while the dashed blue line represents low HER-3 expression. The left panel shows the results for all patients (*n* = 53). The middle panel represents patients with synchronous liver metastasis (*n* = 20), while the right panel shows patients who developed metachronous liver metastasis (*n* = 33).

For the early-stage CRC group, based on the chi-square test, it was determined that there is no association between HER-3 expression and any of the clinicopathological parameters that were examined (Table 1). Moreover, by Kaplan–Meier analyses, no significant correlation was found between HER-3 expression and DFS/OS of early-stage CRC patients, as shown in Fig. 2A. Regarding advanced metastatic CRC group, in primary tumors no significant association was found between HER-3 expression levels and clinicopathological characteristics of CRC patients (Table 2).

**Table 1:** HER-3 status according to clinicopathological features of early-stage CRC patients (*n* = 180).

	HER-3		
Variable	Negative:	Positive:	P*
	*n* (%)	*n* (%)	
Age			
≤60	15 (50.0)	15 (50.0)	
>60	79 (52.7)	71 (47.3)	0.843
Gender			
Male	57 (49.1)	59 (50.9)	
Female	37 (57.8)	27 (42.2)	0.279
T			
T1	6 (66.7)	3 (33.3)	
T2	41 (53.9)	35 (46.1)	
T3	47 (50.5)	46 (49.5)	
T4	1 (50.0)	1 (50.0)	0.912
Grade			
1	9 (60.0)	6 (40.0)	
2–3	85 (51.5)	80 (48.5)	0.597
Tumor Stage			
I	46 (54.8)	38 (45.2)	
II	48 (50.0)	48 (50.0)	0.552
Tumor Location			
Colon	78 (49.7)	79 (50.3)	
Rectum	16 (69.6)	7 (30.4)	0.116

^*^chi-square-test.

**Table 2:** HER-3 status according to the clinicopathological features of advanced metastatic CRC patients (*n* = 53).

Variable	HER-3		*P*
	Low: *n* (%)	High: *n* (%)	
Gender			
Male	12 (48.0)	12 (42.9)	0.786
Female	13 (52.0)	16 (57.1)	
Primary tumor location			
Colon	10 (40.0)	10 (35.7)	
Rectum	15 (60.0)	18 (64.3)	0.783
T status			
1	0 (0.0)	1 (3.6)	
2	2 (8.0)	1 (3.6)	
3	16 (64.0)	19 (67.9)	
4	7 (28.0)	7 (25.0)	0.700
N status			
0	7 (28.0)	6 (21.4)	
1	8 (32.0)	12 (42.9)	
2	9 (36.0)	10 (35.7)	
Not available	1 (4.0)	0 (0.0)	0.622
Liver metastatic tumor nodules			
≤2	14 (56.0)	19 (67.9)	
>2	11 (44.0)	9 (32.1)	0.409
Hepatic resection timing			
Synchronous	12 (48.0)	8 (28.6)	
Metachronous	13 (52.0)	20 (71.4)	0.168
Preoperative CEA (ng/ml)			
≤5	12 (50.0)	12 (54.5)	
>5	12 (50.0)	10 (45.5)	0.777

Interestingly, analyzing the registered CSS rates in this group, we observed that 20 out of 28 patients (71.4%) with HER-3^high^ primary tumors died compared to only 9 out of 25 (36.0%) HER-3^low^ patients. Going to look separately into the SM/MM subgroups, we noticed that among CRC patients with SM liver metastases, 5 out of 8 patients (62.5%) with HER-3^high^ and 7 out of 12 patients (58.3%) with HER-3^low^ in primary tumors died. Otherwise, in patients with MM liver metastases, the CSS rate in the HER-3^high^ expression group was lower than that in the HER-3^low^ expression group (with 75.0% and 15.4% of HER-3^high^ and HER-3^low^ died patients, respectively). Analysis of Kaplan–Meier curves confirmed that patients with HER-3^high^ tumors had a significantly lower CSS rate than patients with HER-3^low^ tumors (*p* = 0.021; Fig. 2B, left panel). Specifically, the significant negative survival value was seen for HER-3^high^ only for CRC patients that developed MM liver metastases (*p* = 0.002; Fig. 2B, middle panel) and not for CRC patients with SM liver metastases (*p* = 0.794; Fig. 2B, right panel).

The role of HER-3 expression as prognostic marker was further confirmed in multivariate analyses of OS adjusted for other prognostic factors. Indeed, as described in Table 3, HER-3 expression resulted to be a significant prognostic parameter influencing survival of CRC patients (HR = 3.3: 95% CI, 1.3–8.3; *p* = 0.011) representing a significant and strong risk factor for death in CRC patients developing MM liver metastases (HR = 64.9; 95% CI, 4.7–886.6; *p* = 0.002).

**Table 3:** Multivariate analysis of HER-3 expression in advanced metastatic CRC tumors.

Variable	HR	95% CI	*P*
CSS in all patients (*n* = 53)			
Gender (male *vs* female)	1.9	0.7–5.5	0.213
Primary tumor location (rectum *vs* colon)	2.9	1.1–7.3	0.025*
Tumor size (T3-4 *vs* T1-2)	2.4	0.3–21.6	0.443
Liver metastatic tumor nodules ( ≤ 2 *vs* > 2)	1.4	0.5–3.9	0.524
Preoperative CEA (ng/ml) ( > 5 *vs* ≤ 5)	1.4	0.6–3.2	0.477
HER-3 (high *vs* low)	3.3	1.3–8.3	0.011*
CSS in patients with synchronous liver metastases (*n* = 20)			
Gender (male *vs* female)	4.1	0.7–24.2	0.115
Primary tumor location (rectum *vs* colon)	1.3	0.3–4.6	0.718
Tumor size (T3-4 *vs* T1-2)	1.7	0.1–4.9	0.989
Liver metastatic tumor nodules ( ≤ 2 *vs* > 2)	5.9	1.0–34.4	0.047*
Preoperative CEA (ng/ml) ( > 5 *vs* ≤ 5)	1.1	0.3–4.0	0.830
HER-3 (high *vs* low)	2.1	0.6–7.0	0.227
CSS in patients with metachronous liver metastases (*n* = 33)			
Gender (male *vs* female)	5.9	1.0–35.5	0.050
Primary tumor location (rectum *vs* colon)	12.5	2.0–78.7	0.007*
Tumor size (T1-2 *vs* T3-4)	100.4	2.0–5102.0	0.021*
Liver metastatic tumor nodules ( > 2 *vs* ≤ 2)	1.3	0.2–7.7	0.793
Preoperative CEA (ng/ml) ( > 5 *vs* ≤ 5)	1.6	0.3–8.7	0.566
HER-3 (high *vs* low)	64.9	4.7–886.6	0.002*

*Statistically significant.

### HER-3 + CRC cells can be targeted by an antibody-drug conjugate

**Fig. 3:**
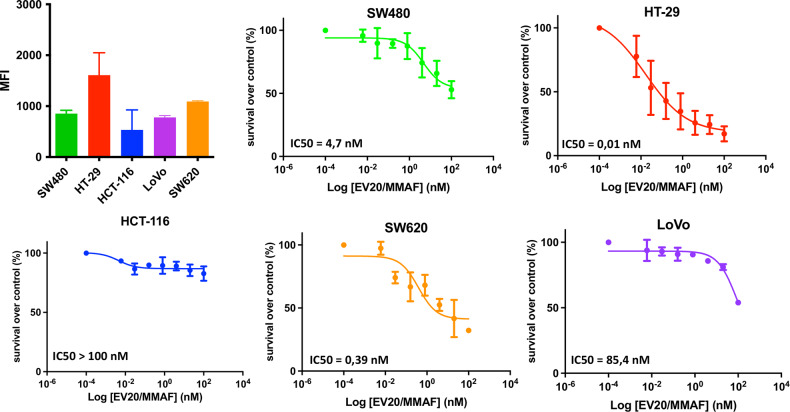
EV20/MMAF ADC in vitro cytotoxic activity. Histogram showing HER-3 surface receptor expression level in colon cancer cell lines analyzed by FACS. MTT assay was performed to evaluate cell killing in colon cancer cell lines treated with increasing doses of EV20/MMAF for 120 h. Percentage of survival over control is represented. Mean ± SD (*n* = 3). IC50 values were calculated using GraphPad Prism 9.0 software.

Furthermore, to establish whether HER-3 may represent a suitable target for targeted therapy in CRC, we evaluate in vitro cell-killing activity of EV20/MMAF, a potent and specific monomethyl auristatin F-based anti-HER-3 ADC developed by our group [19–21]. A panel of five CRC cell lines (HT-29, LoVo, HCT-116, SW620, and SW480) were screened for surface HER-3 expression by FACS analysis and tested for ADC sensitivity to EV20/MMAF. As reported, we observed a correlation between the IC50 and HER-3 expression. Indeed, the cytotoxic activity of the ADC was tested by MTT assay after 5 days exposure, and IC50 values (0.01, 0.39, and 4.7 nM for CRC cell lines expressing higher levels of HER-3) clearly showed that the ADC induces a potent cell-killing activity in a target dependent fashion (Fig. 3).

**Fig. 4:**
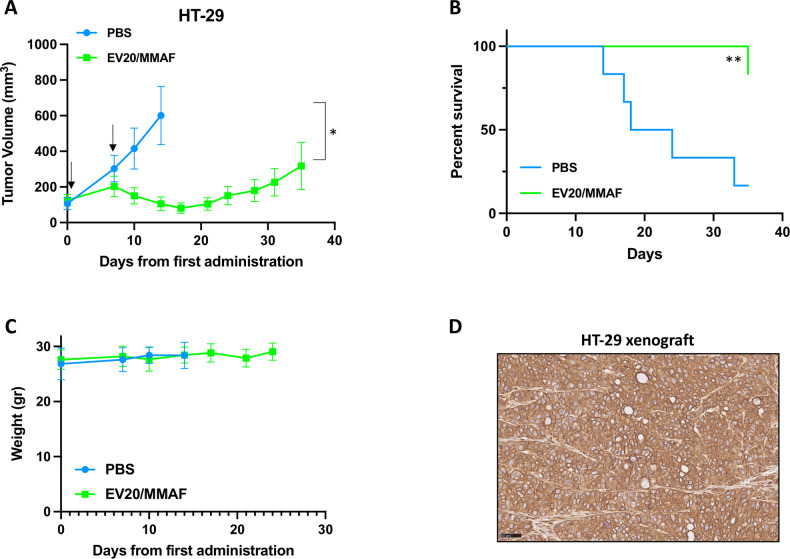
EV20/MMAF therapeutic activity. **A** Growth curves of CD-1 nude mice harboring HT-29 subcutaneous xenografts treated with two weekly administrations (arrows) of vehicle (PBS, *n* = 6) or 10 mg/Kg EV20/MMAF (*n* = 6). *p = 0.01 **B** Kaplan–Meier survival curves analyzed by the Log-rank (Mantel-Cox) Test using GraphPad Prism 8 software **p = 0.009. **C** Graph showing animals’ body weight during the treatments. **D** Representative images of IHC staining for HER-3 expression in HT-29 xenograft.

To validate therapeutic potential of EV20/MMAF in CRC HER-3^high^ HT-29 cell line highly responsive to ADC, which showed lowest IC50 in vitro, it was used as tumor model in a preclinical in vivo study on nude mice. Mice harboring subcutaneous tumors were randomized and divided into two groups (*n* = 6) and treated with EV20-based ADC (EV20/MMAF) once weekly for a total of two administrations at the dose of 10 mg/Kg. Control group received vehicle only (PBS). We observed a strong inhibition of tumor growth in EV20/MMAF treated mice (Fig. 4A) and a significant prolonged survival as evaluated by Kaplan–Meier analysis (Fig. 4B). Notably, no signs of toxicity, in terms of mice weight loss, were observed during the in vivo experiments (Fig. 4C), thus indicating that the ADC is well tolerated in mice. Immunohistochemistry analysis on HT-29 derived xenograft tumor tissue confirmed membrane expression of HER-3 (Fig. 4D).

## Discussion

HER-3 receptor is one of the four members of the human epidermal growth receptors (EGFR/HER) family, which also includes EGFR/*ERBB1*/HER-1, *ERBB2*/HER-2/Neu, and *ERBB4*/HER-4. It’s a well-established paradigm that dysregulation of intracellular signaling controlled by these tyrosine kinase receptors are associated with cell transformation, tumor progression, and metastatic spreading. Indeed, these receptors are overexpressed and/or mutated in a large variety of human cancers [22, 23]. Accordingly, in the last two decades, an huge effort has been produced towards precision medicine, which specifically interferes with ErbB activity [24–26].

Initially, due to its poor kinase activity, HER-3 receptor has not been considered an attractive hit for targeted therapies; however, increasing evidence of this receptor as a key player in regulating escape mechanisms in resistant cells has completely reversed the situation [7, 8].

HER-3 receptor has been extensively investigated in colorectal cancer, both as prognostic factor and therapeutic target. In 2011, a work from Ullrich’s group indicated that patients with intermediate or high HER-3 expression levels had significantly shorter survival times than cases with low HER-3 expression [10]. In a recent set of IHC studies, it’s reported that HER-3 expression correlates with worse prognosis in CRC [11, 12, 27]. In one of these works [11], a cohort of 236 CRC tissues and corresponding lymph node metastases was analyzed, resulting in high HER-3 expression in 75% and 70% of primary tumors and lymph node metastases, respectively. Moreover, it was found that there’s a correlation between HER-3 expression in primary tumors and corresponding lymph node metastases [11]. In a subsequent study, HER-3 overexpression in the primary tumor resulted to be a prognostic factor for OS and DFS [12]. Finally, Styczen et al. [27] showed that HER-3 and HER-2 were overexpressed in colorectal liver metastasis in 75% and 8% of cases, respectively.

From a therapeutic point of view, HER-3-targeted therapies based on the use of monoclonal antibodies, such as patritumab [17] and seribantumab [16], or bispecific monoclonal antibodies against EGFR/HER-3, such as duligotuzumab [18], did not have a great success in terms of clinical benefit in metastatic colorectal cancer patients in clinical trials. Maybe the lack of activity of the naked antibody lies in the fact that inhibition of HER-3 signaling may not be sufficient to inhibit tumor growth, and anti-HER-3 ADC- based therapy could be a turning point. In line with this, a recent work by Koganemaru et al. [14] showed that the use of U3-1402, a potential first-in-class HER-3-targeting ADC, exhibits growth tumor inhibition in human colorectal xenografts models. Our lab has recently developed different ADCs coupling a panel of cytotoxic agents to the humanized anti-HER-3 antibody EV20. As payloads, we have used first the ribosome-inactivating protein (RIP) plant toxin saporin [28] and subsequently tubulin inhibitor (monomethyl auristatin F) or alkylating agent (duocarmycin derivative) [19, 21, 29]. Particularly, auristatin F-based anti-HER-3 ADC, named EV20/MMAF, promoted durable tumor shrinkage in different preclinical models, including metastatic melanoma and breast cancer resistant to HER-2 inhibitors [19, 20].

The aim of the present study was to evaluate HER-3 expression in two different settings of early and advanced metastatic colorectal cancer (CRC) patients. The first one included 180 patients diagnosed with early-stage CRC in absence of lymph nodes or distant metastases (Stage I and Stage II), while the second was obtained from 53 advanced metastatic CRC patients who developed SM and MM liver metastases. We observed a significant increase of surface HER-3 expression in the advanced metastatic primary CRC compared to early-stage tumors both in terms of percentage of positive specimens and mean positive tumor cells in tissues (76,8 versus 100% of positive tissues; 25,7% versus 76,1% of mean positive tumor cells). Also in corresponding liver metastasis membrane expression of HER-3 in cancer cells was elevated (98% of positive metastasis, 63% of mean positive tumor cells), but, disagreeing with Styczen’ study [27], we didn’t reveal increased HER-3 expression compared to primary tumors. Analyzing patients’ clinical outcome, as previously reported [30, 31], we confirmed that metastatic CRC patients with SM liver metastases have a worse prognosis than those with MM liver metastases, indeed Kaplan–Meier analysis showed that the difference registered in CSS rates between SM patients and MM patients was statistically significant (*p* = 0.028; Breslow test) (data not shown). Importantly, while no correlation of HER-3 expression with DFS and OS was found in the early-stage CRC group, on the contrary, when HER-3 expression levels were analyzed in the primary tumors of advanced metastatic CRC group, CSS was significantly lower in HER-3^high^ than HER-3^low^ tumors. In particular, a high HER-3 expression on tumor cell membrane strongly correlates with shorter survival in MM subgroup. Notably, multivariate analysis of CSS confirmed that HER-3 expression represented a significant prognostic parameter influencing survival of CRC patients and represented a significant and strong risk factor for death in CRC patients developing MM liver metastases. Thus, we can speculate that, patients diagnosed with advanced CRC expressing high HER-3 levels in absence of synchronous metastases, will develop metachronous metastatic lesions with increased aggressiveness. Interestingly, similar results were obtained for EGFR trough the IHC analysis of approximately 200 CRC patients [32]. In the perspective of an anti-HER-3 therapy for this subgroup of patients, we perform preliminary in vitro and in vivo therapeutic study with EV20/MMAF demonstrating that HER-3 expressing CRC cells can be efficiently targeted by an anti-HER-3 ADC armed with a tubulin inhibitor.

The limitation of our work is that we were not able to estimate the effect of possible chemotherapy on either HER-3 expression or survival, because the subgroups of CRC patients with different treatments or no treatment were too small. However, nearly all patients with synchronous or metachronous disease received neoadjuvant or adjuvant chemotherapy. In addition, CRC information regarding the patients’ *RAS* and *BRAF* mutation status or the mismatch repair protein status was not routinely available.

In conclusion, our research outlines the importance of assessing HER-3 receptor as a biomarker in advanced metastatic CRC. At the best of our knowledge, this is the first study reporting a prognostic value for HER-3 in patients developing metachronous CRC metastasis. In addition, we confirmed that HER-3^high^ CRC cells can be efficiently targeted in vitro and in vivo by anti-HER-3/ADC EV20/MMAF, thus supporting the notion that the clinical development of such therapy may be promising.

## Materials and methods

### Reagents and cell lines

Antibodies used in the present study were as follows: anti-HER-3 rabbit monoclonal antibody (clone SP-71; Abcam, Cambridge, UK), humanized anti-HER-3 EV20 and its conjugated form EV20/MMAF generated as described [19, 33–35]. HCT-116, HT-29, SW620, and SW480 colon cancer cells were purchased from American Type Culture Collection (Rockville, MD, USA). LoVo cells were purchased from DMSZ (German Collection of Microorganisms and Cell Cultures GmbH). Cells were cultured according to manufacturers’ instructions, propagated less than 3 months after thawing and routinely tested for mycoplasma contamination.

### Patients

The first cohort consisted of 180 patients diagnosed with early-stage CRC (among which 84 of Stage I and 96 of Stage II) without lymph nodes or distant metastases at the diagnosis, already used in a previous study [36]. Out of the total number of patients, 82.2% had colon tumors, while the remaining 17.8% had tumors in the rectum. 116 patients were males (64.4%), and 64 were females (35.6%). The median age of the patients at the time of diagnosis was 69 years. The median follow-up time was 52.5 months. For the second set, a total of 53 advanced CRC patients of an Icelandic population developing liver metastases during the course of the disease were enrolled. Patients and tumor characteristics of the two cohorts of patients are described in Tables 4–5. TNM classification was adopted according to the American Joint Committee on Cancer (AJCC) staging system (8^th^ edition). We considered MM metastasis those diagnosed more than six months after the diagnosis of CRC tumor, while patients diagnosed with liver metastases within 6 months of the primary CRC diagnosis were considered to have SM liver metastases. The mean age at diagnosis was 65.7 years for patients with SM and 65.3 years for CRC patients with MM. Patients’ and tumors characteristics were not significantly different between patients with SM and MM liver metastases (Table 6). The study was approved and reviewed by the Institutional Research Ethics Committee of Iceland (Authorization Nr. VSNb2018060012/03.01). All patients provided written consent for the use of their specimens for research purposes; none were identifiable.

**Table 4:** Clinicopathological characteristics of early-stage (Stage I and Stage II) CRC patients (*n* = 180).

Variable	Value (%)
Age at diagnosis (yr)	
Median	69.5
Range	36–90
Gender	
Male	116 (64.4)
Female	64 (35.6)
Primary tumor location	
Colon	157 (87.2)
Rectum	23 (12.8)
T status	
1	8 (5.0)
2	76 (42.2)
3	93 (51.7)
4	2 (1.1)
Tumor grade	
Well-differentiated	15 (8.3)
Moderately differentiated	152 (84.4)
Poorly differentiated	13 (7.2)
Tumor stage	
I	84 (46.7)
II	96 (53.3)
Clinical outcome	
Disease free	138 (76.7)
Relapse	42 (23.3)

**Table 5:** Clinicopathological characteristics of advanced/metastatic stage CRC patients (*n* = 53).

Variable	Value (%)
Age at diagnosis (yr)	
Median	65
Range	43–87
Gender	
Male	24 (45.3)
Female	29 (54.7)
Primary tumor location	
Colon	20 (37.7)
Rectum	33 (62.3)
T status	
1	1 (1.9)
2	3 (5.7)
3	35 (66.0)
4	14 (26.4)
N status	
0	13 (24.5)
1	20 (37.7)
2	19 (35.8)
Not available	1 (1.9)
Liver metastatic tumor nodules	
≤2	33 (62.3)
>2	20 (37.7)
Hepatic resection timing	
Synchronous	20 (37.7)
Metachronous	33 (62.3)
Preoperative CEA (ng/ml)	
≤5	24 (45.3)
>5	22 (41.5)
Not available	7 (13.2)
Therapy	
Neoadjuvant	14 (26.4)
Adjuvant	33 (62.3)
Palliative	6 (11.3)
HER-3 expression in primary CRC	
Low	25 (47.2)
High	28 (52.8)

**Table 6:** Patients and CRC characteristics according to liver metastasis status.

Variable	Synchronous liver metastasis (*n* = 20)	Metachronous liver metastasis (*n* = 33)	*P*
Gender			
Male	8 (40.0)	16 (48.5)	
Female	12 (60.0)	17 (51.5)	0.582
Primary tumor location			
Colon	10 (50.0)	10 (30.3)	
Rectum	10 (50.0)	23 (69.7)	0.242
T status			
1–2	1 (5.0)	3 (9.1)	
3–4	19 (95.0)	30 (90.9)	1.000
N status			
0	3 (15.8)	10 (30.3)	
1–2	16 (84.2)	23 (69.7)	0.328
Liver metastatic tumor nodules			
≤2	13 (65.0)	20 (60.6)	
>2	7 (35.0)	13 (39.4)	0.779
Preoperative CEA (ng/ml) (*n* = 46)			
≤5	7 (36.8)	17 (63.0)	
>5	12 (63.2)	10 (37.0)	0.134

### Tissue microarray construction and immunohistochemistry

All the primary colorectal cancer surgical specimens and liver metastasis were analyzed at the microscope for finding the best tumor area. After scanning (NanoZoomer XR, Hamamatsu) the hematoxylin and eosin slides of the preselected specimen, markings were done (CaseViewer, 3DHistech) around the preferred area for doing tissue microarray (TMA). Then, 1.0 mm diameter cores from preselected regions of interest of paraffin-embedded tumor samples were assembled in TMA paraffin blocks using TMA Grand Master (3DHistech). The TMAs were sectioned (RM2255, Leica) into 3.5 µm thick sections and transferred to microscope slides for staining.

TMA sections were stained using rabbit anti-HER-3 antibody (Abcam, clone SP-71; dilution 1:100). Antigen retrieval was performed by microwave treatment at 750 W (10 min) in Tris-EDTA buffer (pH 8.0). The anti-rabbit EnVision kit (Agilent, K4003) was used for signal amplification. To exclude unspecific staining, isotype control was included.

### Flow cytometry

For HER-3 surface expression analysis, cells were harvested and labeled with 10 μg /mL of EV20 as a primary antibody for 30 min on ice. After washing, cells were labeled with Alexa Fluor 488-conjugate goat anti-Human IgG Cross-Adsorbed Secondary Antibody (1:300; Molecular Probes, H11013, Life Technologies; Thermo Fisher Scientific, Inc.) for 30 min on ice. Analysis was performed using a FACSCantoII cytometer (BD Biosciences).

### Cytotoxicity assay

For cytotoxic assay, cell lines were seeded into 24-well plates at a density of 5 × 10^3^ cells/well in 500 µL of complete medium; they were treated with increasing doses of EV20/MMAF (ranging between 0.006 and 100 nM) and further incubated for 5 days, as previously described [19]. All experiments were performed in triplicate and the IC50 (Inhibit Cellular Proliferation by 50%) values were calculated using GraphPad Prism 9.0 software (GraphPad Software, Inc., San Diego, CA, USA).

### Therapeutic study

Athymic CD-1 nu/nu female mice (5 or 7 weeks old) were purchased from Charles River Laboratories (Calco, Italy) and maintained at 22–24 °C and relative humidity, under pathogen-limiting conditions as required. Cages, bedding, and food were autoclaved before use. Mice were provided with a standard diet and water ad libitum and acclimatized for 2 weeks before the start of the experiments. Housing and all procedures involving the mice were performed according to the protocol approved by the Institutional Animal Care and Use Committee of the Italian Ministry of Health (Authorization no. 292/2017). Xenografts were generated injecting subcutaneously into the right flank of mice 5 × 10^6^ cells in 200 μl of PBS. When xenografts became palpable (≅100 mm^3^), animals were divided into groups with homogeneous tumor sizes. The treated group received intravenous injections of EV20/MMAF for a total of two administrations at the dose of 10 mg/Kg weekly. Control group received vehicle (PBS) only. The mice weight and the tumor volume were monitored weekly by a caliper and calculated using the following formula: tumor volume (mm^3^) = (length x width^2^)/2. The study was performed in non-blinded modality. A tumor volume of 1 cm^3^ was chosen as endpoint. The Kaplan–Meier method was used to plot the survival curves that were compared using the Log-rank test (Mantel-Cox).

### Statistical analysis

Descriptive statistics were used to describe the distribution of HER-3 expression between the three groups of patients. Differences between groups were evaluated by chi-squared test or Fisher’s exact test for categorical variables, as appropriate. A receiver operating characteristic (ROC) curve was used to predict patient survival to determine the optimal cut-off value for HER-3 expression in primary CRC tissues. Cancer-specific survival (CSS) was defined as the duration from the date of diagnosis until death due to CRC tumor other than other causes. Xenograft growth curves were analyzed using unpaired *t*-test. The Kaplan–Meier method was used to plot the survival curves that were compared using the Log-rank test (Mantel-Cox). Cox’s proportional hazards model tested the association of HER-3 expression with survival. The following covariates were included in the multivariate analysis: gender, primary tumor location, tumor size, number of liver metastatic nodules, preoperative CEA levels, and HER-3 status. All the statistical analyses were performed using the SPSS software (version 15.0; SPSS Inc., Chicago, IL); all tests were two sided, and *p* < 0.05 was considered statistically significant. Sizing of the samples for in vitro and in vivo studies were obtained using the G-Power3.1 software (http://www.gpower.hhu.de/en.html) with a priori analysis type.

## Data Availability

All data generated or analyzed during this study are included in this published article.
